# A New Information-Theoretic Method for Advertisement Conversion Rate Prediction for Large-Scale Sparse Data Based on Deep Learning

**DOI:** 10.3390/e22060643

**Published:** 2020-06-10

**Authors:** Qianchen Xia, Jianghua Lv, Shilong Ma, Bocheng Gao, Zhenhua Wang

**Affiliations:** 1 School of Computer Science and Technology, Beijing University of Aeronautics and Astronautics, Beijing 100191, China; qianchenxia@buaa.edu.cn (Q.X.); slma@buaa.edu.cn (S.M.); gaobocheng@buaa.edu.cn (B.G.); 2 Beijing Insititute of Control Engineering, Beijing 100191, China; zhenhua_07@163.com

**Keywords:** information-theoretic method, advertising conversion rate, LSTM, time series, deep learning, online advertising

## Abstract

With the development of online advertising technology, the accurate targeted advertising based on user preferences is obviously more suitable both for the market and users. The amount of conversion can be properly increased by predicting the user’s purchasing intention based on the advertising Conversion Rate (CVR). According to the high-dimensional and sparse characteristics of the historical behavior sequences, this paper proposes a LSLM_LSTM model, which is for the advertising CVR prediction based on large-scale sparse data. This model aims at minimizing the loss, utilizing the Adaptive Moment Estimation (Adam) optimization algorithm to mine the nonlinear patterns hidden in the data automatically. Through the experimental comparison with a variety of typical CVR prediction models, it is found that the proposed LSLM_LSTM model can utilize the time series characteristics of user behavior sequences more effectively, as well as mine the potential relationship hidden in the features, which brings higher accuracy and trains faster compared to those with consideration of only low or high order features.

## 1. Introduction

With the rapid development of the Internet economy, online advertising has become the main advertising channel. The core of advertising is to discover the most appropriate advertising strategy for the combination of users and the context to increase the overall advertising profit [1]. The advertising performance is reflected by the CVR. This article is mainly focused on the conversion, which is defined as the transformation from users who browse products to users who buy products in e-commerce websites within a certain period of time. That is for retail advertisers who advertise directly to customers. Let CVR = conversions/impression, so a higher CVR can indicate a better conversion effect and more accurate advertisement delivery. The advertising service platform provides sellers with delivery channels and charge fees based on the number of advertising conversions [2]. At present, there are a large number of invalid advertisements that users have no tendencies to click, and sellers waste a large amount of money. If advertisements can be delivered to the targeted users, the conversions can be increased. The CVR affects the ranking results of search engines, reflects the user’s satisfaction with the products, and is one of the important factors in advertising ranking [3]. Therefore, an accurate CVR prediction can increase the advertisement conversion, which can not only optimize the ranking of search engines, but also improve the profit of advertising [4] By analyzing user historical behavior data with the appropriate deep learning models, user information and advertisements are matched to achieve the accurate advertising delivery. However, currently there are three main problems in the CVR prediction: (1) The data scale is too large and sparse to predict the CVR. The historical behavior of users is rich and changeable, simultaneously; the scale of commodities is huge and the statistical characteristics lose meaning. Conversions are based on the impression and the current number of conversions is actually much less than the number of impressions, which lowers the CVR. Therefore, predicting the CVR accurately has become a core task of advertising [5]. The merchant mainly calculates the Cost per Sale (CPS) by the number of goods sold to evaluate the advertising revenue, as shown in the following expression: CPS = N × CVR × ACP, where N represents page views, and ACP represents the fee paid by the advertiser for each conversion. It can be known from the expression that the more accurate the estimation of the CVR, the greater the degree of conversion of advertises placed on the platform, and the higher the benefits. (2) Many fake transactions affect the accuracy of CVR prediction. The top-ranked products are associated with a high CVR. The sales volume is an important factor affecting the ranking. In order to increase the weight of product rankings, some sellers manually manipulate the orders to increase the sales volume of products. The e-commerce platform returns a list of products containing advertisements according to user searches. (3) The time series characteristics of users’ historical behavior are not considered. A series of user operation sequences such as browsing, clicking, and purchasing a product is actually time series data. Taking user behavior as a continuous sequence can better reflect the characteristics of users. Fake orders and user behavior data affect the accuracy of CVR, while the current CVR prediction focuses on data statistics, and rarely considers the time series characteristics and the authenticity of data. All of these increase the difficulty of CVR prediction.

Among the existing CVR estimation methods, typical machine learning methods mainly include Logistic Regression (LR) [6], Gradient Boosting Decision Tree (GBDT) [7], Factorization Machine (FM) [8] and Recurrent Neural Network (RNN) [9]. However, these models merely consider low-order features. Advertising and user behavior data are extremely sparse [10], which is difficult to extract high-order combined features. Feature learning is incomplete and generalization ability is poor. RNN is used to process sequence data [11]. In theory, it supports an infinitely long time series and it can infer the current segments by analyzing the segments in the past. However, with the increase of information volume, the long sequences can cause the gradient to disappear or explode during optimization [12], which makes it impossible to predict conversion efficiently.

The common gradient-based optimization algorithms consist of Stochastic Gradient Descent (SGD) [13], Adaptive Gradient (AdaGrad) [14], and Root Mean Square Propagation (RMSProp) [15,16]. AdaGrad reserves a learning rate for each parameter to improve performance on sparse gradients. RMSProp adaptively retains the learning rate for each parameter based on the average of the recent magnitude of the weight gradient, and has excellent performance on non-steady-state and online training. The Adaptive Moment Estimation (Adam) [17], is an improved algorithm based on SGD, which can achieve the advantages of both AdaGrad and RMSProp.

In response to the problems above, this paper proposes a hybrid model based on the deep neural network, Large Scale Linear Model and Long Short-Term Memory Networks (LSLM_LSTM), according to the user behavior sequence data, product information, and advertisement information of the e-commerce platform, in order to estimate the CVR of advertisements more efficiently and accurately. This model supports the prediction with both high-order and low-order features. Low-level features use a multitasking linear model, which fits nonlinear data and supports artificial feature engineering to effectively identify users’ fake behavior sequences and improve the predictive ability of the model [18,19,20]. The high-order features partially solve the defects of the original RNN model, support the long-term memory of user behavior sequences, and predict users’ purchase intentions more accurately [21,22]. Based on the optimization algorithm Adam, the model learning can be speeded up without increasing computational hardware resources. This paper is divided into four parts. Firstly, the importance of advertising CVR is discussed. Secondly, the advantages and disadvantages of current typical advertising CVR prediction methods are introduced. Thirdly, the LSLM_LSTM model proposed in this paper and the Adam optimization adopted by the model are illustrated. Fourthly, the LSLM_LSTM model through experiments is analyzed. Lastly, the model is summarized.

## 2. Related Work

The advertising CVR prediction can be transformed into a binary classification problem, where X represents the features of model training, and event Y represents whether the user purchases the product. Y∈{0, 1}, where 1 means the product is purchased, and 0 means not purchased. The event is determined by N multivalued discrete features, which are independent in the model by default.

The LR model is a classic classification model that can solve the problem of two or more category classifications. The linear logistic regression is expressed in the following expression, where θ represents weight vector, x represents feature inputs, and $\theta_{0}$ is the offset value.

(1) $$z=\theta^{T}x=\theta_{0}+\theta_{1}x_{1}+\theta_{2}x_{2}+\ldots +\theta_{n}x_{n},$$

The CVR estimation can be transformed into a probabilistic modeling problem. *P* is the predict value. Y=1 means a positive example, and *Y* = 0 means a negative example. The expression of the prediction function is as follows:

(2) $$P\left(Y=1|\theta ;x\right)=\frac{1}{1+e^{-z}},$$

(3) $$P\left(Y=0|\theta ;x\right)=\frac{e^{-z}}{1+e^{-z}},$$

The problem turns to the optimization of finding the maximum value of the log-likelihood function L(θ). The maximum likelihood estimation method is adopted to calculate the loss function. N samples were taken first to estimate model parameters. By calculating the maximum value of L(θ), the optimal solution of the parameter θ is obtained, and the log-likelihood function is as follows:

(4) $$L\left(\theta \right)=\overset{n}{\underset{i=1}{\sum }}y_{i}logh_{\theta }\left(x_{i}\right)+\left(1-y_{i}\right)\;log(1-h_{\theta }\left(x_{i}\right))$$

The CVR was initially trained with a LR model. The advantages of the LR model are simple and with good controllability, which is suitable for solving linear separable problems. The feature combination of the model is the most important part and the learning effect of the feature combination directly affects the accuracy of the CVR prediction. In high-dimensional scenarios, it is easy to reach the upper limit. Feature engineering highly depends on the human, and it is impossible to obtain the nonlinear relationship among features and is easy to be under-fitting, so it requires better feature engineering and produces a lower accuracy.

Compared with using the GBDT prediction directly, the combination of GBDT and LR model witnesses an improvement in AUC by 0.004. Facebook uses the nonlinear model GBDT for feature selection, and the output of GBDT is used as the input of model logistic regression LR. The input of GBDT is continuous data and discrete data separately. By retaining features that appear frequently and filtering out sparse feature values, GBDT effectively solves the problem of feature combination of the LR model, but the disadvantages are still poor memory of historical behavior and poor generalization ability.

The FM model mainly solves the problem of how to combine features in high-dimensional and sparse data. The FM model represents the cross-combined feature by making an inner product of the hidden vector, which improves the nonlinearity of the model. Compared with the linear model LR, there are new improvements in the extraction of low-order and high-order combined features using the model FM. There is no need to manually select features for the cross-feature. However, when there are too many features, the vector has a larger dimension and can only fit a specific nonlinear pattern.

RNN is often used to process sequence data and the model does not rely on manual feature engineering. The neural network improves the generalization ability of the model, but when the matrix is sparse, the feature correlation is poor, and it is easier to perform under-fitting when facing more specific scenarios. The user historical data is actually sequence data, but RNN cannot maintain the coherence of information and will be affected by the short-term memory. During back propagation, the RNN has two problems: Firstly, the gradient is used to update the weight value of the neural network, but the gradient shows an exponential decline over time series; secondly, if a sequence is long enough, it will be difficult to pass information from an earlier time step to subsequent time steps. Table 1 compares the above four models with significant advantages. The traditional machine learning model LR can learn low-order features relatively simply and efficiently. The deep learning-based model has a better learning ability about the combination of high-order features.

**Figure 1:**
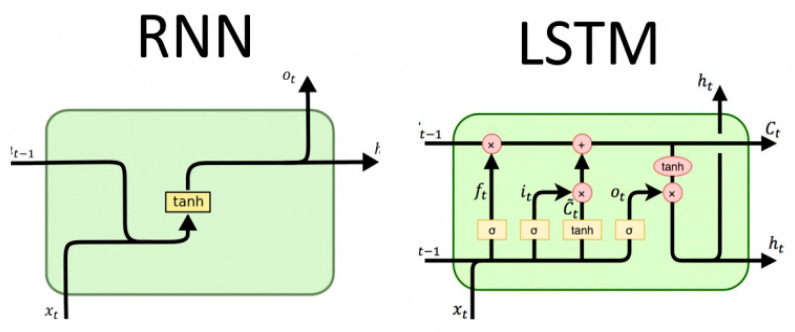
Construction of the recurrent neural network (RNN) and long short-term memory network (LSTM).

Considering the time series characteristics of user behavior data, the studies are based on the LSTM. LSTM is a special RNN, which is suitable for processing and predicting the sequence data. LSTM supports the persistence and memory of the sequence data, which solves the problem of gradient explosion or gradient disappearance in RNN effectively. The cell state is controlled by the gated state in the hidden layer, through which the cell remembers the long-term memory and discards the unimportant information. Each cell contains four kinds of gate structures that interact with each other: Input gate, output gate, forget gate, and cell state. The inner structure of RNN and LSTM is shown in Figure 1. Given sequence $x=\left(x_{1},\;x_{2},\;\cdots ,x_{n}\right)$, the four structures are updated as follows:

(5) $$f_{t}=\sigma \left(w_{f}\cdot \left[h_{t-1},x_{t}\right]+b_{f}\right),$$

(6) $$i_{t}=\sigma \left(w_{i}\cdot \left[h_{t-1},x_{t}\right]+b_{i}\right),$$

(7) $$i_{t}=\sigma \left(w_{i}\cdot \left[h_{t-1},x_{t}\right]+b_{i}\right),$$

(8) $$c_{t}=f_{t}\cdot c_{t-1}+i_{t}\cdot tanh\left(w_{c}\left[h_{t-1},x_{t}\right]+b_{c}\right),$$

(9) $$o_{t}=\sigma \left(w_{o}\cdot \left[h_{t-1},x_{t}\right]+b_{o}\right),$$

(10) $$h_{t}=o_{t}\cdot tanh\left(c_{t}\right),$$

(11) $$y_{t}=\sigma \left(w_{y}h_{t}+b_{y}\right),$$

where w is the weight coefficient, i.e., $w_{i}$ denotes the weight matrix from the input layer to the hidden layer. Subscript *t* denotes moment *t*, and $x_{t}$ denotes the input at moment t, while $y_{t}$ denotes the output y at moment *t*. The forget gate $\;f_{t}\;$ decides what information should be discarded or retained. The input gate $i_{t}$ is used to update the cell status. Each cell has two outputs, $c_{t}$ and $h_{t}$. The cell status $c_{t}$ and the hidden status $h_{t}$ represent the final output of the current cell at moment *t*. The LSTM is trained as follows:

1. The function of $f_{t}$ describes the fact that the previous hidden state $h_{t-1}$ and the current input $x_{t}$ are passed to the σ function at the same time. The closer the output value is to 0 means the closer it is to be discarded, and the closer to 1 means the closer it is to be reserved.
2. The output value $i_{t}$ of the σ function calculates the previous layer hidden state $h_{t-1}$ and the current input $x_{t}$.
3. Input the previous hidden state $h_{t-1}$ and the current input $x_{t}$ to the tanh function to create a new candidate value vector. Finally, $i_{t}$ is multiplied by the output value of tanh. $i_{t}$ that determines which information in the output of tanh is important and should be retained.
4. Add $f_{t}$ and $i_{t}$ point by point, update the new information of the neural network to the cell state $c_{t}$.
5. The next hidden state $h_{t}\;\mathrm{is}\;\mathrm{deteremined}\;\mathrm{by}\;o_{t}$. The hidden state contains the information previously entered. The previous hidden state $h_{t-1}$ and the current input $x_{t}$ are passed to the σ function, and the cell state $c_{t}$ is passed to the tanh function.
6. Multiply the output of tanh by $o_{t}$, and $o_{t}$ to determine the information that the hidden state $h_{t}$ should carry.
7. Use the hidden state $h_{t}$ as the output of the current cell and pass the new cell state $c_{t}$ and the new hidden state $h_{t}$ to the next time step.

The LSTM calculates the adaptive parameter learning rate based on the first-order moment mean, and also utilizes the second-order moment mean of the gradient, which makes the attenuation method of the momentum deviation correction faster than the other two algorithms in the case of a sparse gradient. It is especially applicable for the problems involving high sparse gradients, and with a high calculation efficiency, which is suitable for the estimation of CVR. AUC is an indicator of the pros and cons of a binary classification prediction model. The calculation of AUC is independent of the probability distribution of categories and does not involve the classification cost of different categories. Therefore, when the samples are unevenly distributed in each category in the experiment, AUC can reflect the effect of the classifier.

## 3. Advertising CVR Model Architecture

**Figure 2:**
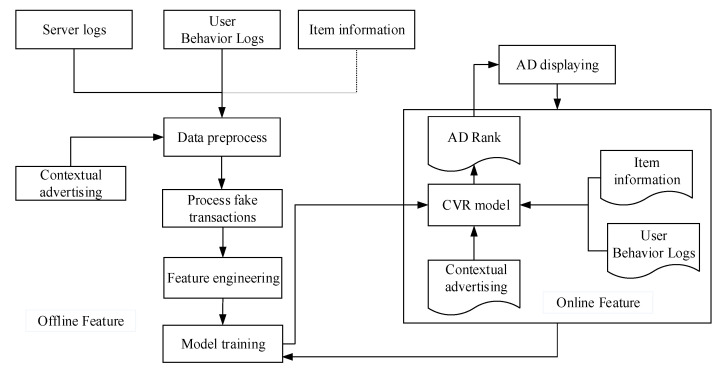
Process of advertising CVR prediction.

CVR is an important metric of advertising quality. The accurate prediction of advertising CVR has become the key to the advertisement delivery technology. The prediction of advertising CVR estimates the probability of users’ purchase behavior based on the click-related users (User), advertising products (Item), advertising context information (AD_Context), stores (Shop), and other information. Conversation = 1 represents that the user purchases the item. Therefore, the probability (PCVR) that a user will make a purchase can be described in a formal definition as follows:

PCVR = P (conversion = 1 | User, Item, AD_Context, Shop). The CVR prediction process steps consists of three steps. Figure 2 shows the detail process of the advertising CVR prediction.

1. Preprocess data on server logs, user behavior logs, product information, and advertising context information to eliminate fake transaction and abnormal data.
2. Perform feature engineering on the preprocessed data. Then, extract features and standardize the input format according to the model requirements.
3. Set and adjust parameters, conduct model training, and get the initial model of the CVR estimation.

### 3.1. Data Cleaning: Removal of Fake Transaction Records

For users, the usual access pattern is to visit the homepage, search for products, browse the product list, click on the product, and enter the product details page. If the user accesses directly through the product URL, then this access is highly likely to be a cheating behavior. Therefore, this paper proposes a strategy to determine the source page for platform access. If the following conditions occur, this access is determined to be a cheating behavior. The access log of the platform server records the information of the http request initiated by the user, which plays a key role in identifying fake transactions.

**Definition** **1.** *(LogData)*. *The access log known as the service request log, describes the basic information of the user requesting services. Define the eight-tuples LogData in Table 2.*

**Definition** **2.** *(ActionLog). The process of purchasing a product is a complex transaction scenario, which mainly includes four types of data: User data, product data, store data, and context data of the user’s searching. The real user purchase behavior is ordered chronologically, followed by the operations of search, browse, click, and purchase. In this operation sequence, there may be multiple steps, such as search, browse, click, return, browse, click, and purchase, but the real and reasonable sequence of operations must include the sequence of search, browse, click, and purchase.*

The user’s behavior sequences can be described as a two-tuples S:: = (LogData, ActionLog).

Assume that there are n operations, and the user’s behavior sequences is defined as S = $\left(S_{1},\cdots ,S_{n}\right)$. ActionLog is illustrated in Table 3.

**Definition** **3.** *(Fake Transactions). Fake Transactions are mainly composed of three main kinds of components: Abnormal refer data, abnormal consumption behavior, and abnormal operation sequence. Abnormal refer data is about the problems of users’ http request. The abnormal consumption behavior consists of the abnormal behavior, which is extremely different from the user’s historical behavior and the abnormal product status, which are mainly reflected in the transaction frequency, transaction amount, and transaction cycle. They are illustrated in Table 4.*

### 3.2. Feature Preprocessing

The CVR estimation has a large number of numerical types of features, which can be divided into continuous features and category features. In order to prevent some features from affecting the data too profoundly, a standardized method is adopted for continuous numerical features, so that the value can be in [0, 1]. One-hot encoding is performed on the category features, and the original features are converted into high-dimensional and sparse vectors after encoding. By keeping the original feature expression ability as much as possible, this paper uses the Embedding method to compress high-dimensional vectors into low-dimensional vectors. During the training of deep neural networks, feature vectors are constantly updated, and the similarity between features can be calculated in a multidimensional space, which improves the speed of training and reduces the memory overhead.

### 3.3. Deep Learning Network Based on the LSLM_LSTM Model

**Figure 3:**
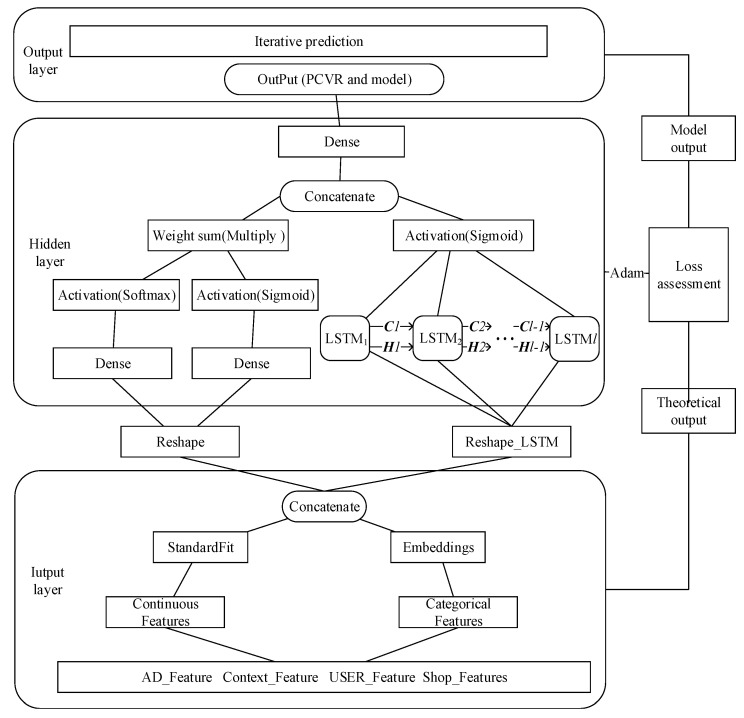
Advertising CVR prediction framework based on the large scale linear model and long short-term memory networks (LSLM_LSTM).

The behavior of users considering whether purchasing the items is continuous during a certain period of time. The information of user behaviors which happens closer to the purchasing moment are more likely to embody the users’ shopping intents. The essence of LSTM is a sequence of relationships between the current output and previous information. By retaining previous information in the current node, it supports the time series input in parallel as separate variables or features. Considering the characteristics of a multivariate time series, the framework of the LSLM_LSTM model proposed in this paper is shown in Figure 3. The model consists of five modules: Input layer, hidden layer, output layer, network training layer and model prediction. The input layer processes the original input data to meet the network input requirements. The layer processes continuous and discrete data separately. In order to handle both high-order features and low-order features at the same time, the LSLM fits the nonlinear low-order feature data, and the LSTM constructs a neural network to fit user behavior sequence feature data. The prediction results of these two parts are concentrated to the next layer by an ordinary weighting method. The LSLM and LSTM are jointly trained. The same back propagation of the gradient affects all parts of the model at the same time and the output layer obtains the prediction results. The classification results are compared with the sample labels, and the cross-entropy loss function is calculated. The network training uses Adam to optimize the weights.

Different groups of people have clustering characteristics that people have similar preferences in the same cluster. For example, people with a strong purchasing ability tend to buy items with a high price. The LSLM divides the feature space into γ regions, in which the sequences fit a linear model according to the features and output the joints of the weighted sum result. Nonlinear models are obtained from the sparse data, and the feature region fragmentation joint with linear model fitting makes the data size that the model can handle larger. This reinforces the nonlinear representation of the model and makes convergence easier. Expression (12) is the probability function.

(12) $$P\left(Y=1|x\right)=\sigma \left(W_{lslm}^{T}\cdot g\left(\overset{\gamma }{\underset{j=1}{\sum }}\mu \left(u_{j}^{T}x\right)\sigma \left(w_{j}^{T}x\right)\right)+W_{lstm}^{T}f\left(w_{l}^{T}x\right)+b\right),$$

$W_{lslm}^{T}$ and $W_{lstm}^{T}$ are the parameters in the weight matrix, γ is the number of features’ partitions. A larger γ demonstrates the stronger fit ability of the model. $u_{j}$ and $w_{l}$ represent the cluster weight parameters which determine the division of the space. $w_{j}$ represents the classification weight which determines the prediction in the space. σ denotes the sigmoid activation function; g represents the probability function, and μ represents the classification function softmax. The prediction framework of the advertisement CVR based on LSLM_LSTM is shown in Figure 3.

The loss function in this paper is defined as follows:

(13) $$logloss=-\frac{1}{N}\overset{N}{\underset{1}{\sum }}\left(y_{i}\log\left(p_{i}\right)+\left(1-y_{i}\right)\log\left(1-p_{i}\right)\right),$$

where *N* is the total number of test samples, and $y_{i}$ is a binary variable whose value is 0 or 1. It denotes the label of the *i*-th sample, and $p_{i}$ is the probability that the model predicts that the label of the *i*-th sample is 1. The smaller the value, the higher the accuracy of the conversion rate prediction. The larger the value of AUC and the smaller the value of Log Loss, the better the classifier effect.

The training data of CVR contains both classification and continuous features, which are extremely sparse and high-dimensional. In order to reduce the feature dimensions, the LSLM_LSTM model makes the category features embed during the data preprocessing stage to map the data to a low-dimensional space. The output of the embedding layer replaces the original category features as the first hidden layer of the LSTM network that makes the input vectors compress into low-dimensional dense vectors. The output of the LSLM are jointed with the LSTM network to establish a new network. The model uses its multilayer network structure to learn the nonlinear correlation among the features layer by layer. It is not enough to transfer the complete data set once in the neural network, and we need to input the data set several times in the same neural network. The number of model weight updates is the number of training iterations, which performs a gradient descent to calculate the loss and outputs the model as well as the prediction. That is the iteration prediction.

### 3.4. Model Network

The samples of behavior sequences are the multivariate time series. Define the user behavior sequences $S=\left\{s_{1},s_{2},\cdots ,s_{n}\right\}$ with a length n which is the size of the data set, and divide the data set into a training set and a test set with m, then the training set is $S_{tr}=\left\{s_{1},s_{2},\cdots ,s_{m}\right\}$, while the test set is $S_{te}=\left\{s_{m+1},s_{m+2},\cdots ,s_{n}\right\}$, where m<n, and m, n∈R.

We process the features according to whether the features are discrete or continuous. That is the continuous features will be standardized and the discrete features will be embedded. Normalize the continuous features and embed the discrete features. The category feature $f_{c}$ is embedded into $f_{c}^{\prime }$ and $f_{t}^{\prime }=\frac{\left(f_{t}-f_{mean}\right)}{f_{std}}$ is used to transform the continuous one-dimensional feature $f_{t}$ to $f_{t}^{\prime }$ with the mean $f_{mean}$, the standard deviation $f_{std}$ of the features. Thus, the fit training dataset is $S_{tr}^{\prime }=\left\{s_{1}^{\prime },s_{2}^{\prime },\cdots ,s_{m}^{\prime }\right\}$. Assume the feature dimension is β, and the $Features=\left\{f_{1},f_{2},\cdots ,f_{\beta }\right\}$, then the processed feature set is denoted as $Features^{\prime }=\left\{f_{1}^{\prime },f_{2}^{\prime },\cdots ,f_{k}^{\prime }\right\}$. The embedding involves the input dimension $l_{in}$ representing the count of different values in the data set, and the output dimension $l_{out}$ which defines the size of the output vector dimension of the embedding layer after dimension reduction. The LSTM contains the input of the sequences samples which has the length l, where each one has a time step q, and the output is a single time step. The gate structures of the LSTM network are in the hidden layer that contains the loop bodies of the LSTM and is connected in chronological order. The ellipsis in the hidden layer indicates the hidden neurons and the number of LSTM layers. Actually, the behavior sequence at moment t−1 is utilized into the model to predict the behavior at moment *t*. Defining the input of LSTM as $X=\left\{X_{1},X_{2},\cdots ,X_{l}\right\},X_{t}=\left\{s_{t}^{\prime },s_{t+1}^{\prime },\cdots ,s_{m+t-l-1}^{\prime }\right\},\;1\leq t\leq l;\;t,\;l\in R$, the theoretical output is $Y_{lstm}=\left\{Y_{1},Y_{2},\cdots ,Y_{l}\right\},Y_{t}=\left\{s_{t+1}^{\prime },s_{t+2}^{\prime },\cdots ,s_{m+t-l}^{\prime }\right\}$, and the corresponding output is $P_{lstm}=\left\{P_{1},P_{2},\cdots ,P_{l}\right\},P_{t}$ denotes the output of $P_{lstm}$ at moment t, where $P_{t}=\mathrm{LSTM}\left(X_{t},C_{t-1},H_{t-1}\right)$, $C_{t-1},\;H_{t-1}$ denotes the cell status and hidden status at moment t−1. $P_{lslm}=$LSLM($X_{1},X_{2},\cdots ,X_{l}$), P = $\sigma \left(W_{lslm}^{T}\cdot P_{lslm}+W_{lslm}^{T}\cdot P_{lstm}\right)$, two parts share the same so the output layer concatenates $P_{lstm}$ and $P_{lslm}$ to generate the model output P, which is PCVR in Section 3. The LSLM_LSTM model memorizes the cell state with the necessary information and transmits it in the neuron. The calculation of the model is concatenated with two parts, which is applied with the Adam optimization to minimize the loss, the learning rate η, and updates the model weight. $\mathrm{LSLM}\_{\mathrm{LSTM}}_{\mathrm{net}}$ represents the joint model of LSLM blocks and the LSTM hidden layer network. The algorithm is described in Table 5.

The training process involves the feature data input, hidden layer, output layer and network training. The input layer mainly performs feature processing and conversion.

## 4. Experimental Analysis

The model proposed targets how to effectively and efficiently process large-scale sparse data, while enhancing the generalization ability of the model, so the training of the two parts can be performed simultaneously. In this section, the model proposed in Section 3 is discussed in detail and verified based on the prediction.

### 4.1. Experimental Data and Analysis

**Figure 4:**
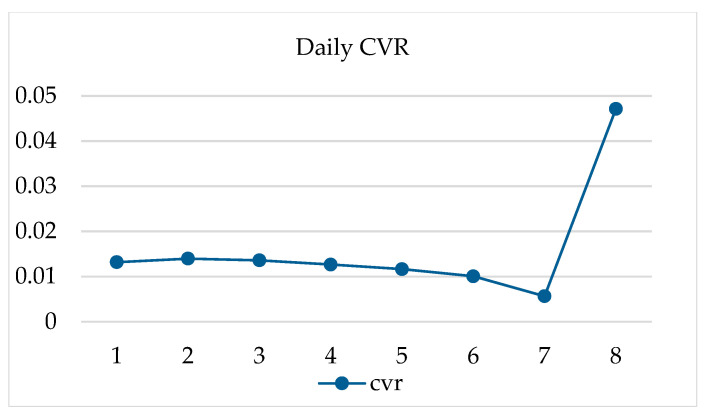
Daily advertising conversion rate.

The datasets used in the experiment are the public dataset of Alibaba Advertising in 2018 and the Retailrocket purchase recommendation dataset with 1,407,580 users and 2,756,101 behavior records. The original Alibaba dataset contains 11 million user transaction records, and the sample labels are 1 and 0, which represent positive and negative samples of the conversion. As shown in Figure 4, the CVR from the first day to the seventh day is relatively stable, remaining at about 1.2%. However, the CVR on the eighth day increased significantly to 4%.

The methodology is validated as follows. Firstly, the preliminary data is cleaned according to the fake transaction data cleaning rules of Definition 3 to remove noncompliant data. According to the analysis, the fake transactions of the data accounted for 15%, including the sudden increase in clicks in a short time, multiple clicks at the same time, and abnormal user behaviors such as the unreasonable sequence of user operations, and these data were removed as required. At the same time, it is found that the distribution of positive and negative samples of the data was extremely uneven. In order to prevent too many negative samples from reducing the prediction accuracy, the dataset was filled by adding positive samples to keep the positive and negative samples at a ratio of 7:3. In real data, we should adjust the ratio of positive and negative data for model learning in the entire feature space. It is also possible to randomly remove some data, such as high-frequency and low-frequency sparse data. After that, we actually divide the data into N parts to balance the data, using N trainers and finally average the prediction result. Therefore, the model will work with the same high accuracy.

In addition to fake data, there are some illegal data in the dataset, so there are mainly three types of data cleaning tasks:

Data type check: If a field has a value that does not belong to the field type, it can be determined that the value is an abnormal value, which needs to be removed or replaced with a default value.

Special character clean-up: Handle special characters in logs and standardize data field separators.

Outlier filtering: Analyze whether the data range is unreasonable, according to business rules, or statistics analysis. Statistics include maximum, minimum and average values. If the data distribution follows a normal distribution, the measured value is considered abnormal if the deviation from the mean exceeds three standard deviations.

After the data is cleaned, the numerical types are divided into continuous features and category features. According to the formula for normalizing continuous numeric features in Section 3.3, the values are standardized to [0, 1]. One-hot encoding is performed on the category features, and the original features are converted into high-dimensional and sparse vectors after encoding. Without losing the original feature expression ability as much as possible, the features are embedded to compress high-dimensional vectors into low-dimensional vectors. During the training of deep neural networks, the feature vectors are constantly updated, and the similarity between features can be calculated in a multidimensional space, which improves the speed of training and reduces memory cost.

**Figure 5:**
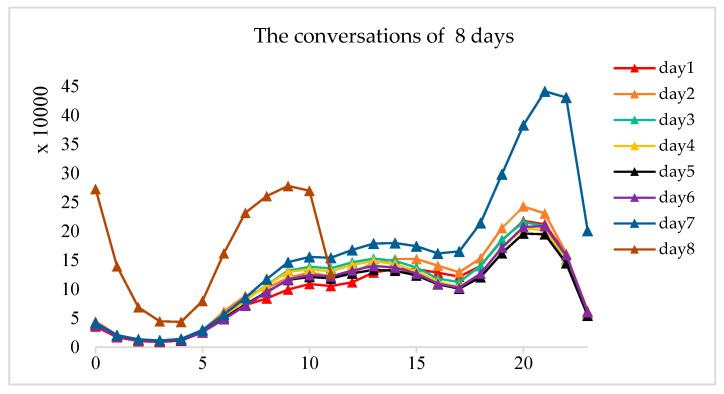
Conversions per day.

The dataset is divided into a training set and a test set at the ratio of 3:1. Mark the date of the data as 1st to 8th. As shown in Figure 5, the advertising conversion volume from the first day to the seventh day has maintained the same pattern, and the amount of conversion increased significantly from 6:00 pm of the seventh day to 2:00 am of the eighth day. Therefore, it can be known that the eighth day is a special holiday, and the data to be predicted on the seventh day is in the second half of that of the special holiday. In order to ensure that the data follow the same distribution, the data is divided into two parts. The first part is for modeling the user behaviors in the first six days and the rest of the part is the second part. The first part of the user interaction data contains the stable user and product, and stores historical information so we can use these data to characterize the model.

### 4.2. Feature Selection and Analysis

Except for the emission of the fake data, the processing of data also includes transforming discrete features into one-hot features and the normalization of continuous features. In this paper, the features of key information such as users, products, stores and context are divided as follows:

- Origin characteristics: The user star rating, product price level, product sales level, product collection times level, product display times level, store evaluation number, store praise rate, store service attitude score, store description match score and other characteristics.
- Historical statistical characteristics: The clicks count, conversions, and CVRs of first-order features and second-order cross-features of users, products, and brands in the previous seven days to describe the products, brands, shops and categories on the last day.
- User behavior characteristics: The characteristic subject is the user, and characterizes the behavior of the user from all sides, including how many times the user clicked in the day, the user’s delta time between this click and the last click, whether the user interacted with the product for the first time, whether the user is repurchasing, the amount of the products the user clicked under the same query, the price order of the product by the user under the same query, etc.
- Embedding characteristics: The product has multiple attributes. The number of the product ID is too large, so this feature is large-scaled and sparsely. The dimension after one-hot is too large. By embedding the feature according to the relationship between the product and the attributes, the product attribute pair is established. Eventually, the vectors of the products and attributes are received respectively and can be inputted directly into the model.

### 4.3. Evaluation

In this paper, Accuracy (AUC) and log loss are used to evaluate the training results of the model. Considering the unevenness of positive and negative sample distribution of the dataset, AUC is suitable for the evaluation of advertising CVR prediction.

### 4.4. Experimental Environment

The experiment running environment contains a software environment and hardware environment. The configuration is shown in Table 6.

The more complicated the model, the more time and space complexity is required. Even the phenomenon of over-fitting will occur. In order to optimize the model results, the experiment carried out a lot of parameter adjustment work. The parameters involved mainly include: The number of LSTM layers, the steps, the dimensions of the output vector, the dimensions of the input and output vectors of the embedding layer, the optimization method of the model, and the learning rate. It was found through experiments that the model learning rate and optimization methods have a relatively large impact on the experimental results. The experimental parameters are shown in Table 7.

### 4.5. Experimental Results and Analysis

#### 4.5.1. Experiment 1

To study the prediction effects of different models, the experiment is performed on the models LR, GBDT, FM, LSTM and LSLM_LSTM. The parameters of the LSLM_LSTM are determined through multiple experiments. The experiment cleans the data according to the fake transaction with the principle proposed in Section 3 previously. In addition, the continuous features and category features are processed in line with the input requirements of the model.

In order to minimize the loss, we define different learning rates which affect the speed of training and the accuracy of prediction results. The number of iterations is 100, and the batch size is 5000. The model discards the activation of some neurons to make the model more robust and prevent over-fitting. The dropout is 0.2, that is, 20% of the neurons are not the output. After feature engineering, there are 1.55 million feature categories, and the embedding process spends a long time. Let the output dimension of embedding be 36 dimensions, which greatly reduces the feature dimension and deeply improves the training efficiency and accuracy while retaining the features.

**Figure 6:**
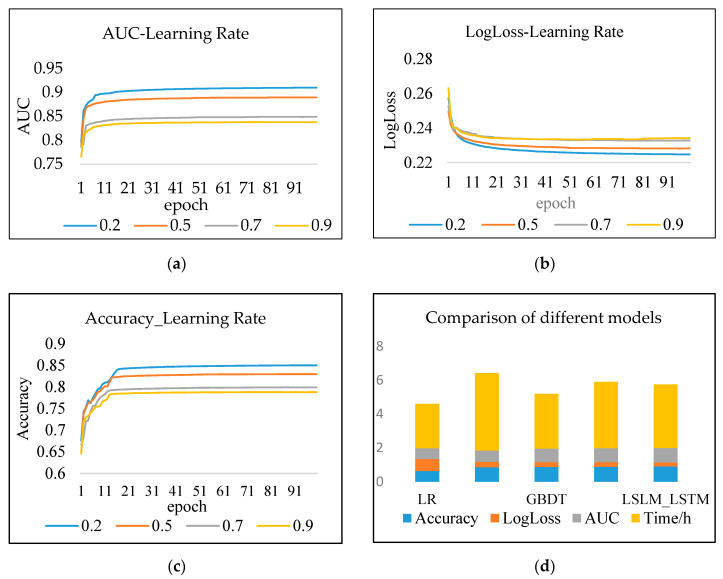
Comparison of results at different learning rates. (**a**) Accuracy (AUC)-Learning Rate; (**b**) LogLoss-Learning Rate; (**c**) Accuracy-Learning Rate; (**d**) comparison of different models.

To obtain the optimal AUC, the LSLM_LSTM model utilizes different parameter values to iterate 100 times. It can be seen from Figure 6 that when the learning rate is large, the model converges fast, but the effect is not as good as that when the learning rate is small. When the learning rate is 0.2, there is probably a stable AUC in the 11th epoch, which is better than the result with a large learning rate.

Results analysis: As seen in Table 8: (1) The prediction effect of the deep model is better than the traditional shallow model, and the effect of LR fitting large-scale sparse data is poor. The model’s AUC and log loss basically converge in approximately 11 iterations. FM is superior to LR while GBDT and LSTM is better than FM in the experiment. (2) The AUC and log loss of LSLM_LSTM are better than the LR model and LSTM model with a single structure. Compared with the LR model, its accuracy is increased by 43.29%, and its AUC is increased by 27.5%, and its log loss is reduced by 73%. Compared with the LSTM model, the accuracy is increased by 2.81%, and the AUC value is increased by 2.72%, and its log loss is reduced by 12.4%. (3) The more layers of the LSLM_LSTM hybrid neural network, the higher the complexity of the training time cost, which is more than the single structure. The prediction efficacy has been greatly improved. The model designed balances the two aspects that enhance the generalization ability and improve the memory ability of nonlinear low-order models.

#### 4.5.2. Experiment 2

The dataset Retailrocket is a user behavior dataset collected by a real-world e-commerce website. The dataset contains 1,407,580 users and 2,756,101 behavior records with a series of user clicks and purchases. The product information includes category, brand, price, etc. The user information includes user group, gender, age, consumption level and city level. The behavior logs are mainly the user’s operations such as browsing, carting, and purchase with a CVR value of 0.82%. It is a popular dataset in the field of recommender systems with implicit feedback and suitable to verify the proposed algorithm, as shown in Table 9.

**Figure 7:**
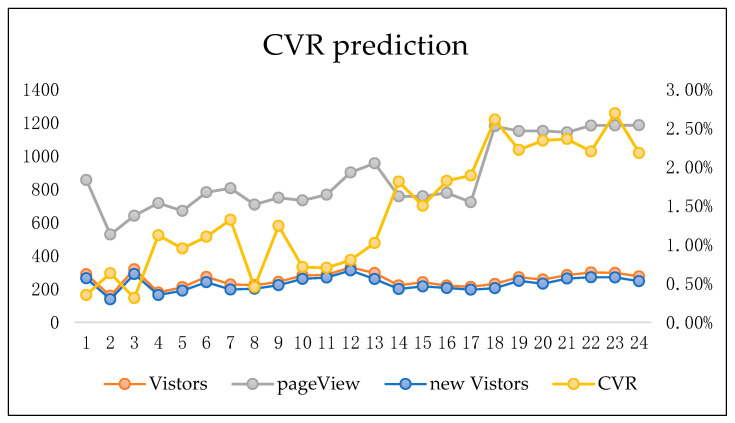
An implement result comparison of the proposed CVR prediction model in 24 days.

In order to verify the impact of the estimated conversion rate of ads, the method proposed in this article is applied on a real commerce website. Figure 7 is the visualization of the CVR values for 24 days. The left indicator shows the numbers of Visitors, pageView and new Visitors. We can know the CVR value in the right indicator. In the first 12 days, data was brought out with the LR model, and data in the following 12 days was brought out with the proposed model in this article. From the perspective of the number of visitors, page views and new visitors, it can be seen that after applying the proposed LSLM_LSTM model, the average conversion rate comparison between the previous 12 days and the following 12 days, with the help of the model, the advertisers obtained a number of significantly increased CVR by 5.3%. Even if the new visitors number still remains at the same level, which means a more accurate advertising to users. It proves that the proposed model is effective in more accurate targeted advertising, which leads to more page views and higher CVR.

Results analysis: As seen in Table: (1) The prediction of the proposed model on Retailrocket as well as the prediction on the Alibaba dataset is better than the traditional shallow model, and the effect of LR fitting large-scale sparse data is poor. FM is superior to LR while GBDT and LSTM is better than FM. (2) Compared with the LR model, its accuracy is increased by 34.03%, and its AUC is increased by 26.3%, and its log loss is reduced by 58.9%. Compared with the LSTM model, the accuracy is increased by 2.81%, and the AUC value is increased by 1.12%, and its log loss is reduced by 10.3%. (3) The higher complexity of the training time cost. The proposed model has a similar result on the two datasets. In addition, the learning speed is 1/2 times of the training speed on the Alibaba dataset for its smaller size. The AUC, log loss, and accuracy are all as well as the result on the Alibaba dataset with a lower value. However, in comparison of the five models, they reflect the same regular pattern.

The CVR prediction is a complicated process. The conversion rate estimation involves a large number of user behaviors, product attributes and advertising features. The method of combining models in parallel can further improve the prediction effect. The shallow models are superior in the combination of low-order features. These models have a good performance, especially the LR model has the simplest architecture and the fastest calculation speed. The LSTM model and the model proposed in this paper are superior in the combination of higher-order features. Such deep models can extract hidden information in the data in more detail.

## 5. Conclusions

For the issue of Internet online advertising CVR prediction, this paper proposes a LSLM_LSTM model based on large-scale sparse data, which technically maps the user’s behavior and preferences. The higher-order feature model integrates the low-order nonlinear features, and end-to-end complete training, which enhances the association among the feature vectors. The experiments prove that the LSLM_LSTM model can learn the complex relationship among the large sparse features and has been proven to present more accurate results. Through cleaning the fake data and considering the time series characteristic of data, this model makes the classification task of CVR prediction more accurately and efficiently. The CVR is an indicator used to measure the possibility that ads will be converted by users. If the CVR can be accurately estimated, it will bring great benefits for both users and advertisers. Users will get more accurate advertising information; advertisers will get more user conversions. By applying the algorithm to the online system, user conversion has been significantly improved and a certain effect has been achieved, indicating that the idea of using the algorithm proposed in this paper to predict the conversion rate is appropriate.

Advertising data in many application scenarios exist in the form of text, which does not belong to numbers and cannot be utilized in the proposed model directly. In order to solve this problem, natural language processing is required to process the data. In the future, it will be considered to establish an advertisement conversion rate estimation model that integrates both text features and digital features.

## Figures and Tables

**Table 1:** Comparison of conversion rate (CVR) prediction models.

Models	Pre-Train	High-Order Feature Combinations	Low-Order Feature Combinations	Feature Engineering	Support Time Series
LR	No	No	Yes	No	No
GBDT + LR	Yes	Yes	Yes	No	No
FM	No	No	Yes	Yes	No
RNN	Yes	Yes	No	Yes	Yes

**Table 2:** Definition of LogData.

• LogData:: = (Time, UserAddr, ReferPage, ServerAddr, ProtType, ReqResURL, ReqSize, Status, UAgent)	
• Attribute	• Description
• Time	• Time:: = DD/MM/YY:H:M:S TimeZone
	• user access time.
• UserAddr	• IP address which the user can access from.
• ReferPage	• the source page from which the user links to the current page
• ServerAddr	• ServerAddr:: = (ServName, ServIP, ServPort)
	• the host providing the service, ServName is the server name. ServIP denotes the IP address. ServPort denotes the access port.
• Status	• Status:: = SuccessCode|(ErrCode)
	• the reque.st status, SuccessCode denotes succeeded code, ErrCode indicates failure code.
• ReqResURL	• ReqResURL:: = (URL, Params)
	• the request URL and request parameters.
• ProtType	• ProtType:: = (“Http”, ProtVer, ReqMethod)
	• the type of the request protocol, ReqMethod:: = {“Get”, “Post”};
	• ProtVer represents the protocol version and ReqMethod denotes the request methods.
• UAgent	• the information about the client browser.

**Table 3:** Definition of ActionLog.

• ActionLog:: = (User, UserAddr, Item, Shop, Context, Action, Time)	
• Attribute	• Description
• User	• User:: = (userID, gender, age, occupation, starLevel, consumAbility)
	• user’s occupation, gender, age, the higher the star level value, the higher the level of the user; ConsumAbility denotes consumption ability, the larger the value, the stronger the purchasing ability.
• Item	• Item:: = (itemID, itemCategory, itemProperty, price, brand, review, LBS, price, sales, collecLevel, PVLevel);
	• item’s ID, category, attribute collection, brand, price, sales, delivery location, and review rank in the shop;
	• collecLevel: Rank of the times the product has been a favorite;
	• PVLevel: Rank of the times the product has been clicked, the larger the value, the more the clicks.
• Shop	• Shop:: = (shopID, shopReview, shopRate, servScore, delivScore, descripScore, Location)
	• shopID: the shop ID;
	• shopReview: review rank;
	• shopRate: the positive reviews from users;
	• servScore: the service rating;
	• delivScore: the delivery service rating from users;
	• descripScore: the rating of the matching description;
	• Location: the city of the shop.
• Context	• Context:: = (ctxID, ctxTime, ctxPageID, ctxCategory, predictProperty)
	• the context displayed after the user’s searching for items with detailed information of page, category, property, and searching time.
• Behavior	• Behavior:: = query | scan |click | buy
	• users’ behavior categories in the website.

**Table 4:** Definition of fake transactions.

• Fake Transactions	
• Category	• Description
• Abnormal refer data	• User access without a refer page or not a media source, or the product is directly reached through a link, instead of clicking through the product list interface.
	• ReferPage = Null | NoAdSource, where Null denotes a null value, and NoAdSource denotes that it is not a media source.
• Abnormal consumption behavior	• 1. The account is new and frequently traded within a short period of time;
	• 2. User’s historical transaction volume is small, and the amount is low, but frequent and high amount transaction records are recorded in a short time;
	• 3. The user browses the products or clicks in the same time interval and the time for browsing the products is short;
	• 4. The product is generated by browsing PV or click behavior, and a large number of displays were generated on the same product within a short period of time. The number of clicks, purchases, exposures, and clicks skyrocketed at a certain point in time and most of the products purchased come from the same IP;
	• 5. The merchant will require the users to collect the product when they purchase. Therefore, the collected product is an important criterion to judge whether it is a fake transaction. Take the time of the last transaction record as a node, and count the ratio of the total number of transactions of this product to the total number of people who collect this product. If the ratio is high, it is a fake transaction.
• Abnormal operation sequence	• A click is directly generated without viewing the advertising product, or the product has already been purchased before it has been exposed.

**Table 5:** LSTM_LSLM algorithm.

Algorithm 1 LSTM_LSLM Algorithm
Input: User behavior sequences S and S length n, training set $S_{tr}$ length m, test set $S_{te}$, time steps q, the learning rate η, the input dimension $l_{in}$, the output dimension $l_{out}$, the length of the input sequences samples l, the partitionsnumber of LSLM γ. Output: The model with trained weights and PCVR P 1: function LSTM_LSLM ($S,\gamma ,m,n,q,\eta ,l_{in},l_{out},l$) 2: get $S_{tr},S_{te}\;from$ S by m 3: ${\mathrm{AS}}_{tr}^{\prime }\leftarrow \mathrm{fit}\_\mathrm{transform}\left({\mathrm{AS}}_{tr}\right)$ 4: $f_{c}^{\prime }\leftarrow \mathrm{embedding}\left(f_{c}\right)$ 5: get $X,Y{\;\mathrm{from}\;\mathrm{AS}}_{tr}^{\prime }$ By m,output_len 6: set γ regions in LSLM 7: create ${\mathrm{LSTM}}_{cell}\;by\;C_{t},H_{t}$ 8: connect LSLM_LSTM By $m,\;steps,l\;and{\;\mathrm{LSTM}}_{cell}$ 9: initialize $\mathrm{LSLM}\_{\mathrm{LSTM}}_{net}$ 10: for each step in 1:steps 11: P=LSLM_LSTM(X) 12: Loss ← cross_entropy 13: update $\mathrm{LSLM}\_{\mathrm{LSTM}}_{net}$ by Adam with loss and η 14: end for 15: return$\;\mathrm{LSLM}\_{\mathrm{LSTM}}_{net}^{\prime }$, P, AUC, Accuracy, Loss of $\mathrm{model}.\mathrm{evaluate}\left(S_{te}\right)$ 16: end function

**Table 6:** Experimental environment.

	Operating System	Cent OS
Software	Language	Python 3.7
	Framework	TensorFlow
	Development Tools	Anaconda, Spider, MongoDB
Hardware	Memory	64 G
	Hard disk	2T
	Processor	Intel(R) Xeon(R) CPU E7-4809 v3 @ 2.0 GHz

**Table 7:** Experimental parameters.

Parameters	Value
Learning rate	0.2, 0.5, 0.7,0.9
Optimization	Adam
Embedding output	36
LSTM Layers	3
Steps	4
Dropout	0.2
Batch size	5000

**Table 8:** Comparison of prediction results of different models on the Alibaba dataset.

Models	Accuracy	LogLoss	AUC	Time/h
LR	0.624022	0.70321	0.622113	4.63
FM	0.852734	0.329425	0.653719	7.59
GBDT	0.856727	0.283173	0.773762	6.25
LSTM	0.873646	0.268724	0.793508	6.92
LSLM_LSTM	0.898497	0.249352	0.824377	6.76

**Table 9:** Comparison of prediction results of different models on the Retailrocket dataset.

Models	Accuracy	LogLoss	AUC	Time/h
LR	0.614051	0.69319	0.642838	1.31
FM	0.823018	0.339507	0.646786	2.46
GBDT	0.825789	0.307183	0.769365	1.58
LSTM	0.847632	0.289261	0.789631	1.96
LSLM_LSTM	0.854077	0.257158	0.812156	1.72
